# Some Account of Tipioca; and of the Species of Ipecacuanha Found near Rio Janeiro

**Published:** 1788

**Authors:** 


### VIII. *Some Account of Tipioca; and of the Species of Ipecacuanha found near Rio Janeiro.*

WE have written the firft of thefe names as we find it written by Pifo, who in his work *de Indiæ utriufque re naturali et medica**, has given a particular account of the fubftance which, within thefe few years only, has been imported into this country under the name of *Tapioca.*

* Folio, Amftelædami, 1658, p. 116.

 This

This writer, ſpeaking of the mandihoca, ſays, "Ex hoc frutice expreſſo manat liquor, *mani-* "*puera* Barbaris dictus, qui vaſi infuſus poſt "duas horas fundo adhæreſcit; ex quo . . . "fit farina quam cremorem de *Tipioca* vocant. "Ex aqua farinæ, in fundo ſubſidente, bolos "quoque conficiunt, *Tipiocato* dictos, optimi "ſaporis. Tum Gummi quoddam, ſeu po- "tius amylum ex ea fit, atque eidem uſui in- "ſervit." This agrees with the following account of this ſubſtance, which, with the permiſſion of Sir Joſeph Banks, Bart. Preſident of the Royal Society, we have extracted from a letter he has lately received from Arthur Phillip, Eſq. Governor of New South Wales, and which is dated at Rio Janeiro, Auguſt 31ſt, 1787. "I have had — ſays Governor Phillip — ſome "Tapioca made at this place. The water "that drains from the mandioca, or caſſada, "has a very great ſediment, which is a fine "white powder, and which is dried for hair "powder; or prepared in a different manner, "and made into Tapioca. This is all I can "learn on the ſubject, and I believe all you "wiſhed to know."

This was not the only inquiry relative to natural hiſtory made by Governor Phillip during his ſtay at Rio Janeiro. Among other things, he has ſent to Sir Joſeph Banks ſpecimens of Ipecauanha; from which it appears, that both the grey and the brown ſpecies of it are found in the neighbourhood of that place. This piece of intelligence will be intereſting to botaniſts; for although two ſpecies are mentioned by Piſo in the work * juſt now referred to, yet it ſeems to have been pretty generally ſuppoſed by later writers on the materia medica, that only one of them, viz. the brown, is found in Braſil.

